# Comparison of Anesthetic and Surgical Outcomes Between General Anesthesia and Dexmedetomidine‐Based Sedoanalgesia in Dacryocystorhinostomy Surgery With Bispectral Index Monitoring

**DOI:** 10.1155/anrp/6650312

**Published:** 2025-11-04

**Authors:** Utku Sağlam, Sertaç Argun Kıvanç, Nermin Kelebek Girgin

**Affiliations:** ^1^ Department of Anesthesiology and Reanimation, Faculty of Medicine, Bursa Uludağ University, Bursa, Turkey; ^2^ Department of Ophthalmology, Faculty of Medicine, Bursa Uludağ University, Bursa, Turkey

**Keywords:** bispectral index, dacryocystorhinostomy, dexmedetomidine, general anesthesia, sedoanalgesia

## Abstract

**Background:**

This study aimed to compare general anesthesia (GA) and sedoanalgesia (SA) with dexmedetomidine in terms of anesthetic and surgical outcomes in dacryocystorhinostomy (DCR) under bispectral index (BIS) monitoring.

**Methods:**

In this prospective randomized controlled study, 44 adult patients (≥ 18 y) were divided into two groups. Standard GA was induced in the GA group (*n* = 22), and dexmedetomidine‐based SA in the SA group (*n* = 22). BIS monitoring was performed in all patients. Hemodynamic parameters, amount of intraoperative bleeding, duration of anesthesia and surgery, postoperative pain scores, complications, and patient and surgeon satisfaction were recorded.

**Results:**

The hemodynamic parameters, amount of intraoperative bleeding, duration of anesthesia and surgery, postoperative pain score, complications, and patient and surgeon satisfaction were similar between the two groups. Patient satisfaction was significantly higher in the SA group (*p* = 0.033).

**Conclusions:**

This study suggests that SA with dexmedetomidine accompanied by BIS monitoring can be an alternative to GA in selected patients undergoing external DCR surgery, because the anesthetic and surgical processes are similar to those of GA, and patient satisfaction is higher than that with GA.

**Trial Registration:**

ClinicalTrials.gov identifier: NCT05397301

## 1. Introduction

Primary acquired nasolacrimal duct obstruction is a common disease of the nasolacrimal duct, the gold standard procedure for which is external dacryocystorhinostomy (DCR) surgery. External DCR can be performed under general anesthesia (GA) and local anesthesia (LA) with or without sedation [1, 2]. Because patients who undergo DCR are generally elderly with comorbidities, the desire to avoid the risks of GA has increased the tendency toward sedoanalgesia (SA) and LA use in the last 2 decades [3–5]. The use of LA in DCR reduces intraoperative bleeding, analgesia requirements, and incidence of postoperative nausea and vomiting (PONV) [4–6].

Maintaining a stable systemic arterial blood pressure (BP) for intraoperative hemostasis during DCR is important. While the patient’s heart rate (HR) and BP control are easier with GA, the adequacy of LA alone is debatable. It has been suggested that applying LA together with sedation is more effective in controlling HR and BP. In addition, sedation is thought to improve the outcomes of DCR in terms of patient and surgeon comfort and intraoperative complications. It is also shown that SA is a safe and efficient method for reducing intraoperative bleeding and length of hospital stay [7, 8].

Dexmedetomidine is a highly alpha‐2 selective adrenergic receptor agonist drug, whose use in anesthesia practice is increasing day by day. Despite its dose‐dependent sedative, anxiolytic, and analgesic effects, it does not cause respiratory depression [9, 10]. Objective monitoring is the ideal method for measuring sedation levels. Bispectral index (BIS) monitoring is an electroencephalography parameter widely used in clinical practice to determine anesthesia depth during anesthetic or sedative drug administration [11, 12]. To the best of our knowledge, no previous study has investigated the use of dexmedetomidine and BIS monitoring in DCR.

Therefore, the aim of this study was to compare GA and dexmedetomidine‐based SA with BIS monitoring in terms of hemodynamic parameters, amount of surgical bleeding, postoperative complications (bleeding, pain, and PONV), and patient and surgeon satisfaction in DCR surgery.

## 2. Methods

After obtaining approval from the faculty ethics committee (number: 2020‐2/11, date: 5th February 2020) and written informed consent from the participants, 44 adult patients (≥ 18 years old), with American Society of Anesthesiologists (ASA) Physical status I‐II‐III, who were undergoing unilateral primary DCR surgery, were included in this prospective randomized controlled study.

The exclusion criteria were as follows: ASA > III, body mass index (BMI) > 40, allergy to drugs used in the study, alpha‐2 receptor agonist therapy, drug and/or alcohol abuse, serious liver or kidney failure, mental or psychiatric disorders, and refusal to participate in the study.

Routine monitoring with electrocardiography, noninvasive arterial BP, and peripheral oxygen saturation (SpO2) was performed in the operating room. In addition, all patients were monitored using a BIS (Aspect A‐2000, USA), and the anesthesia and sedation depths were monitored.

Patients were randomly assigned in a 1:1 ratio to either the GA or SA group using a computer‐generated randomization sequence. Allocation was concealed in sequentially numbered, sealed opaque envelopes. On the day of surgery, the envelope for each patient was opened by the supervising anesthesiologist, who was unaware of the study protocol and randomization sequence, and the assigned anesthetic technique was communicated to the anesthesiologist assigned to that case. Postoperative patient satisfaction was assessed by the ward nurse on duty, who was blinded to the group allocations, to minimize potential subjective bias in satisfaction scores.

In the GA group, GA was induced with midazolam (0.04 mg kg^−1^), lidocaine (1.2 mg kg^−1^), fentanyl (2 μg kg^−1^), thiopental sodium (4 mg kg^−1^), and rocuronium (0.6 mg kg^−1^) intravenously (IV). Following endotracheal intubation, anesthesia was maintained with sevoflurane and 50/50% oxygen/air to achieve an end‐tidal carbon dioxide of 35–40 mmHg. Supplementary doses of fentanyl 25–50 μg kg^−1^ IV were administered according to the patient’s needs. Owing to the routine GA protocol at our hospital, a prophylactic antiemetic drug (metoclopramide 10 mg, IV) was administered to all patients in the GA group. In the SA group, midazolam (0.04 mg kg^−1^) and dexmedetomidine (1 µq kg^−1^ 10 min bolus, followed by 0.6 µq kg^−1^ h^−^ continuous infusion) were administered IV for sedation, and pethidine 1 mg kg^−1^ was administered intramuscularly for analgesia. The BIS score was maintained within the range of 60–80 in the SA group and 40–60 in the GA group. Antiemetics were not routinely administered in the SA group but were administered intraoperatively only when clinically indicated at the discretion of the attending anesthesiologist.

All DCR operations were performed by an experienced surgeon (SAK), and infiltration anesthesia was administered on the incision line by the surgeon in both groups. Methylprednisolone (1 mg kg^−1^ IV) was administered to all patients as part of a routine surgical protocol.

Hemodynamic variables (systolic BP [SBP], diastolic BP [DBP], mean arterial BP [MAP], and HR), SpO_2_, and BIS values were recorded preoperatively and at 5, 10, 20, 30, and 40 min after the surgical incision.

The suction chamber was cut at the end of the surgery, and the total intraoperative bleeding was measured using an injector. In addition, blood from sterile sponges and cotton swabs was added to this measurement. Intraoperative complications, such as canalicular damage and cerebrospinal fluid leak, surgical duration (from surgical incision to skin closure), and anesthesia duration (from monitoring of the patient in the operating room until transport of the patient to the recovery room), were recorded.

Postoperative pain was evaluated with scores of 0–10 using the visual analog scale (VAS) (0, no pain; 10, worst pain imaginable). Before surgery, all the patients were instructed on the use of the VAS in a short session. VAS scores were recorded in the recovery room and at 30 min and 1, 2, 4, 8, and 12 h postoperatively. If the VAS score was ≥ 4, paracetamol (1 gr, IV) was administered. The patients were observed for side effects (nausea and vomiting), need for antiemetic drugs, and surgical complications (hematoma, ecchymosis, epistasis, inflammation, edema, and failure of tube placement) during the postoperative period. The degree of patient and surgeon satisfaction with anesthesia was assessed using a five‐point Likert scale (1–5 points: very dissatisfied, dissatisfied, scarcely satisfied, satisfied, and very satisfied).

### 2.1. Statistical Analysis

The distribution of the continuous variables was assessed using the Shapiro–Wilk test. Variables that were normally distributed are reported as mean ± standard deviation, while variables that were not normally distributed are reported as median (interquartile range [IQR]).

Comparisons of preoperative measurements between the groups were conducted using either the independent samples *t*‐test for normally distributed variables or the Mann–Whitney *U* test for non‐normally distributed variables. For time‐dependent measurements (5, 10, 20, 30, and 40 min after incision), percentage changes were calculated based on the preoperative values. Group comparisons for these percentage changes were similarly analyzed using the independent samples *t*‐test or Mann–Whitney *U* test, depending on the distribution characteristics.

The amount of intraoperative bleeding and changes in VAS scores (pre‐ vs. postoperative) were not normally distributed and were compared between groups using the Mann–Whitney *U* test. Satisfaction scores (both surgical team and patient) were also analyzed using the Mann–Whitney *U* test due to non‐normal distributions.

Categorical variables, including intraoperative antiemetic use, presence of PONV, rate of antiemetic use, and rates of intraoperative and postoperative complications, were analyzed using either the Pearson chi‐square test or Fisher’s exact test. The choice between these tests was based on the expected frequency assumption: Pearson’s chi‐square test was used when all expected cell counts were 5 or greater, while Fisher’s exact test was applied when at least one expected cell count was below 5, ensuring validity of the statistical inference for small sample sizes.

A Type I error level of 5% was considered statistically significant. All statistical analyses were performed using IBM SPSS for Windows, Version 23.0 (IBM Corp., Armonk, NY, USA).

## 3. Results

The mean age of the patients was 59.56 ± 13.94 years, and 35 (79.5%) of them were women. The demographic characteristics, duration of surgery, and duration of anesthesia were similar between the two groups (Table 1).

**Table 1:** Demographic and surgical characteristics of the patients.

	SA group (*n* = 22)	GA group (*n* = 22)	*p* value
Age (year) (mean ± SD)	59.90 ± 12.10	59.22 ± 15.85	0.873
Gender (*n*, %)			> 0.99
Female	17 (77.3%)	18 (81.8%)	
Male	5 (22.7%)	4 (18.2%)	
BMI (kg/m^2^) (mean ± SD)	29.78 ± 3.98	30.03 ± 5.35	0.861
ASA score (*n*, %)			0.397
I	11 (50%)	7 (31.8%)	
II	9 (40.9%)	14 (63.6%)	
III	2 (9.1%)	1 (4.5%)	
Surgery time (min) (mean ± SD)	55 ± 17.25	57.04 ± 11.61	0.647
Anesthesia time (min) (mean ± SD)	79.77 ± 16.07	85.45 ± 14.46	0.225

*Note:* SA, sedoanalgesia.

Abbreviations: ASA, American Society of Anesthesiologists; BMI, body mass index; GA, general anesthesia.

No significant differences in SBP, DBP, MAP, or HR were observed between the two groups during the preoperative period. In the GA group, there was a significant intraoperative decrease in SBP and MAP at all measurement timepoints, as well as in DBP at 5, 10, 20, and 40 min, compared to the preoperative values (all *p* < 0.05). No significant changes were observed in these parameters in the SA group. In addition, there were no significant changes in the HR in either group during the intraoperative period. The hemodynamic changes during anesthesia are shown in Table 2.

**Table 2:** Systolic and diastolic blood pressure changes (mean ± SD).

	Group SA (*n* = 22)	Group GA (*n* = 22)	*p* value
*SBP (mmHg)*			
Preoperative	154.81 ± 22.99	159.22 ± 26.93	*0.562*
Intraoperative 5 min	122.68 ± 23.19	104.68 ± 16.06	
Intraoperative 10 min	117.50 ± 23.01	94.22 ± 14.20	
Intraoperative 20 min	113.13 ± 17.50	97.81 ± 16.62	
Intraoperative 30 min	113.00 ± 19.41	97.13 ± 14.89	
Intraoperative 40 min	117.36 ± 19.23	93.90 ± 13.13	
%Δ5 min^∗^ ← preoperative	%↓ 19.53 ± 16.98	%↓ 32.61 ± 13.92	**0.008**
%Δ10 min ← preoperative	%↓ 22.78 ± 18.01	%↓ 39.04 ± 14.04	**0.002**
%Δ20 min ← preoperative	%↓ 25.85 ± 13.00	%↓ 36.75 ± 15.03	**0.014**
%Δ30 min ← preoperative	%↓ 25.76 ± 14.92	%↓ 36.58 ± 17.25	**0.032**
%Δ40 min ← preoperative	%↓ 22.89 ± 15.22	%↓ 38.74 ± 16.42	**0.002**

*DBP (mmHg)*			
Preoperative	91.13 ± 11.49	90.81 ± 11.72	*0.928*
Intraoperative 5 min	76.00 ± 13.17	65.59 ± 11.12	
Intraoperative 10 min	70.54 ± 15.55	57.45 ± 9.04	
Intraoperative 20 min	70.5 (13.75)	60 (11.5)	
Intraoperative 30 min	67.31 ± 12.38	59.09 ± 10.53	
Intraoperative 40 min	73 (16.5)	52.5 (15.25)	
%Δ5 min^∗^ ← preoperative	%↓ 16.25 (21.26)	%↓ 24.61 (12.26)	**0.027**
%Δ10 min ← preoperative	%↓ 22.00 ± 18.15	%↓ 35.87 ± 11.82	**0.005**
%Δ20 min ← preoperative	%↓ 21.69 ± 13.41	%↓ 32.09 ± 13.32	**0.014**
%Δ30 min ← preoperative	%↓ 22.48 (20.61)	%↓ 33.67 (21.02)	0.074
%Δ40 min ← preoperative	%↓ 19.76 ± 12.19	%↓ 36.47 ± 16.16	**< 0.001**

*MAP (mmHg)*			
Preoperative	114.09 ± 14.58	115.95 ± 17.90	0.707
Intraoperative 5 min	91.95 ± 15.29	78.95 ± 12.48	
Intraoperative 10 min	84.72 ± 16.84	69.72 ± 10.16	
Intraoperative 20 min	84.81 ± 15.20	73.95 ± 13.92	
Intraoperative 30 min	84.36 ± 14.72	71.77 ± 12.81	
Intraoperative 40 min	88.40 ± 13.37	68.31 ± 1114	
%Δ5 min^∗^ ← preoperative	%↓ 18.52 ± 15.14	%↓ 30.44 ± 14.18	**0.010**
%Δ10 min ← preoperative	%↓ 24.61 ± 18.08	%↓ 38.36 ± 13.24	**0.006**
%Δ20 min ← preoperative	%↓ 24.78 ± 14.66	%↓ 34.76 ± 14.95	**0.031**
%Δ30 min ← preoperative	%↓ 25.36 ± 13.64	%↓ 35.83 ± 18.21	**0.037**
%Δ40 min ← preoperative	%↓ 21.75 ± 13.00	%↓ 38.86 ± 17.29	**0.001**

Note: SA, sedoanalgesia; MAP, mean arterial blood pressure. The bold values indicate statistically significant values.

Abbreviations: DBP, diastolic blood pressure; GA, general anesthesia; SBP, systolic blood pressure.

^∗^Percentage change in the measurements during the intraoperative period compared to those during the preoperative period.

The preoperative BIS scores were similar between the groups. BIS scores were maintained within the target range in both groups during the intraoperative period.

No additional analgesic was required in either group during the intraoperative period.

The amount of intraoperative bleeding was 79.5 (±158.5) mL in the GA group and 44 (±116.5) mL in the SA group (*p* = 0.078).

The VAS scores at all timepoints and the postoperative analgesic requirements did not differ significantly between the GA and SA groups (Table 3).

**Table 3:** VAS score changes (interquartile range: IQR).

VAS score (0–10)	Group SA (n = 22)	Group GA (n = 22)	*p* value
Postoperative 30 min	1 (2)	1 (2)	0.883
Postoperative 1 h	1 (2)	0 (2)	
Postoperative 2 h	0 (1)	0 (1)	
Postoperative 4 h	0 (0)	0 (0.25)	
Postoperative 8 h	0 (0)	0 (0)	
Postoperative 12 h	0 (0)	0 (0)	
%Δ1 h ← 0–30 min	%↓ 1 (2)	%↓ 1 (2)	0.971
%Δ2 h ← 0–30 min	%↓ 1 (1.25)	%↓ 1 (1)	0.803
%Δ4 h ← 0–30 min	%↓ 1 (2)	%↓ 1 (1.25)	0.546
%Δ8 h ← 0–30 min	%↓ 1 (2)	%↓ 1 (2)	0.971
%Δ12 h ← 0–30 min	%↓ 1 (2)	%↓ 1 (2)	0.971

*Note:* SA, sedoanalgesia.

Abbreviations: GA, general anesthesia; VAS, visual analog scale.

Postoperative nausea, vomiting, and antiemetic use were similar between the two groups (Table 4).

**Table 4:** Surgeon and patient satisfaction (interquartile range: IQR), intraoperative antiemetic use, postoperative nausea–vomiting ratio, and postoperative antiemetic and analgesic use (*n*, %).

	Group SA (*n* = 22)	Group GA (*n* = 22)	*p* value
Surgeon satisfaction^∗^	5 (1)	5 (0.25)	0.566
Patient satisfaction^∗^	5 (0.25)	4 (1)	**0.033**
Postoperative nausea–vomiting	1 (4.5%)	1 (4.5%)	> 0.99
Postoperative antiemetic use	1 (4.5%)	1 (4.5%)	> 0.99
Postoperative analgesic use	1 (4.5%)	1 (4.5%)	> 0.99

*Note:* SA, sedoanalgesia. The bold value indicates statistically significant value.

Abbreviation: GA, general anesthesia.

^∗^Likert scale (1–5 points: 1 = strongly disagree, 2 = disagree, 3 = neither agree nor disagree, 4 = agree, and 5 = strongly agree).

Laryngospasm occurred in one patient (4.5%) in the GA group following extubation. Moreover, no surgical complications were observed in any patients included in this study.

Surgeon satisfaction was comparable between the two groups. By contrast, patient satisfaction was significantly higher in the SA group (Table 4).

## 4. Discussion

In this study, GA and SA with BIS monitoring were compared in terms of anesthetic and surgical outcomes such as hemodynamic parameters, intraoperative bleeding, postoperative complications (pain, PONV, etc.), and patient and surgeon satisfaction with external DCR surgery. Both techniques were effective, but patient satisfaction was higher in the SA group.

Although GA is accepted as the gold standard for external DCR, there is growing interest in LA and SA. Both techniques are preferred, especially in older patients because of their poor health [1, 3–8]. Intraoperatively, hemodynamic parameters must be within the normal range to protect end‐organ perfusion and provide hemostasis. Tuladhar et al. [7] detected that sedation was superior to the LA‐only method in terms of SBP, DBP, and HR in external DCR surgery. Chaume et al. [8] showed that LA with sedation can provide hemodynamic stability similar to GA in external DCR surgery and demonstrated that in the LA with the sedation group, SBP, DBP, and HR remained stable from the incision to 30 min postoperatively. In our study, there was a decrease in the intraoperative BP in both groups compared to the preoperative values. Although the decrease in BP was greater in the GA group, BP was stable in both groups during the intraoperative period. HR also decreased in both groups during the intraoperative period compared to during the preoperative period but remained within the normal range.

Controlling intraoperative bleeding is important in external DCR. Ensuring bleeding control provides a better surgical field of view and increases surgical success [13]. Çiftçi et al. [4] determined that the average intraoperative blood loss in external DCR surgery was 16.93 ± 3.23 mL in the GA group and 8.98 ± 2.79 mL in the LA group. Tuladhar et al. [7] reported that intraoperative blood loss was lower in the sedation group than in the LA‐only group during DCR. Kasaee et al. [14] observed that intraoperative blood loss was similar between the GA (62.6 ± 59.9 mL) and LA (50.5 ± 42.6 mL) groups during DCR surgery. In our study, intraoperative blood loss was found to be similar between the GA (79.5 [158.5] mL) and SA (44 [116.5] mL) groups. This may be attributed to the administration of LA at the incision site in both groups, the alpha‐2 agonistic effects of dexmedetomidine, sedation, and the use of anesthetic drugs under BIS monitoring, which maintained stable hemodynamics.

External DCR surgery may cause mild‐to‐moderate postoperative pain. Short‐acting narcotic analgesic drugs, local anesthetic infiltration at the wound site, and nonsteroidal anti‐inflammatory drugs or paracetamol can be used for analgesic management [1, 2, 4, 6]. Knezevic et al. [6] compared the LA and GA methods and found that pain levels were significantly higher in the GA group. In addition, pain localization was evaluated in this study, and no significant differences were observed between the two groups. Tuladhar et al. [7] found that postoperative pain scores were significantly lower in the sedation group than in the LA group after external DCR surgery. In our study, pain was assessed over 12 h during the postoperative period, and the VAS scores were low and similar in both groups. Only one patient in each group required an analgesic. These results support the view that GA and SA provide adequate postoperative analgesia.

Complications related to anesthesia and surgery may occur during DCR. The possible surgical complications include hematoma, ecchymosis, epistaxis, inflammation, edema, and failed tube placement. No surgery‐related complications were observed in our study. The most common anesthesia‐related complications include airway management, postoperative pain, and PONV [4, 15, 16]. Harissi‐Dagher et al. [1] observed that, compared to patients in the GA group, those in the LA group required significantly less antiemetic medication within the first 4 h postoperatively (*p* = 0.03). Çiftçi et al. [4] reported that the incidence of nausea was 7.6% in the GA group and 1.6% in the LA group. The incidence of vomiting was 5.4% and 1.3% in the GA and LA groups, respectively. The highest rates of these two complications were detected at 5 min postoperatively. In the study by Knežević et al. [6], PONV was particularly observed within the first 1‐2 h. In this series, PONV occurred more frequently than in the Çiftçi et al. series but less frequently than in the Harissi‐Dagher et al. series. Richard et al. [15] reported that nausea and vomiting were extremely rare in patients who underwent DCR surgery under GA and received prophylactic antiemetics. Mohammed et al. [16] followed up 90 patients who underwent DCR surgery under GA without prophylactic antiemetics for 24 h and reported rates of nausea and vomiting of 35% and 23%, respectively. Since antiemetics are part of the routine GA protocol at our hospital, a prophylactic antiemetic drug was administered to all patients in the GA group and three patients in the SA group. One patient from each group experienced PONV. Despite the absence of routine prophylactic antiemesis, the SA group had PONV rates similar to those in the GA group. We concluded that this may be attributed to the antiemetic effects of dexmedetomidine, and that the routine administration of methylprednisolone by the surgical team could have contributed to this outcome.

Surgeon and patient satisfaction is important for all surgical interventions. Tuladhar et al. [7] found that surgical comfort was high with LA alone and with the addition of sedation because of the less bloody surgical field. Chaume et al. [8] reported that LA combined with sedation yielded high levels of patient and surgeon satisfaction. In their prospective study involving 34 cases, surgeon satisfaction was found to be excellent in 88.3% of cases, with scores ≥ 7/10 (range: 5–10). Regarding patients, 91.3% expressed satisfaction with the quality of anesthesia, and 94.2% were satisfied with their level of comfort during surgery. In our study, we used a Likert scale to assess satisfaction and found that surgical satisfaction was similar in both groups, whereas patient satisfaction was higher in the SA group. We believe that this may be related to the lack of discomfort associated with intubation and extubation and the faster recovery from dexmedetomidine.

Dexmedetomidine has become an appealing sedative agent in ophthalmic surgeries, particularly cataract surgeries, because of its ability to stabilize hemodynamics and suppress reflex tachycardia and sympathetic responses [17–19]. In this study, we used dexmedetomidine as a sedative agent under BIS monitoring and found that it was effective for sedation during DCR.

Our study has several strengths and limitations. Its strengths include the use of BIS monitoring for DCR and its prospective randomized controlled design. The limitations of this study include a relatively small sample size, which was influenced by institutional constraints due to the specialized nature of DCR surgery and additional restrictions on elective procedures during the coronavirus disease 2019 pandemic. Moreover, the involvement of a single surgeon and anesthesiologist responsible for perioperative evaluations contributed to limitations in blinding. In addition, the routine prophylactic use of antiemetic drugs in the GA protocol at our institution and the administration of intraoperative antiemetics to only three patients in the SA group limited the effective comparison of PONV outcomes between the two groups. Furthermore, the administration of intramuscular meperidine in the SA group may have influenced postoperative pain score assessments. Nevertheless, the similar VAS scores between the groups and high patient and surgeon satisfaction levels suggest that our method was effective in providing analgesia and ensuring patient comfort.

## 5. Conclusions

We found that SA accompanied by BIS monitoring was similar to GA in terms of hemodynamic parameters, intraoperative bleeding, and complications, but superior to SA in terms of patient satisfaction. Considering these findings, we consider SA with dexmedetomidine under BIS monitoring as a viable alternative to GA for external DCR surgery in appropriately selected patients.

## Conflicts of Interest

The authors declare no conflicts of interest.

## Funding

No funding sources were involved in data collection, analysis, interpretation, trial design, patient recruitment, or any aspect pertinent to the study.

## Data Availability

The data that support the findings of this study are available from the corresponding author upon reasonable request.
